# Fellow-Workers and the Roll of Honour

**Published:** 1915-06-12

**Authors:** 


### ROUND THE WORLD.

[The Editor invites Early Information and Contributions Marked "Fellow-Workers."]

### Fellow-Workers and the Roll of Honour.

Second-Lieutenant Roy Fazan, who was killed on May 9, was a Middlesex Hospital student, and only twenty-three years of age. A keen Territorial, he was given a commission in his battalion (5th Royal Sussex) a few months before mobilisation, and after training went abroad in February. The youngest son of Dr. C. H. Fazan, of Wadhurst, Sussex—himself a "Middlesex" man—Mr. Roy Fazan was educated at Epsom College, where he was a member of the O.T.C.

The county town of Lancaster has lost one of its medical men by the death of Captain Frank Miller Bingham, who had been at the Front for some time as a combatant officer, and lost his life on May 22. A St. Thomas's Hospital student, who qualified by taking the diploma of the Conjoint Board in 1900, the late Captain Bingham accepted a commission in the King's Own Royal Lancaster Regiment (5th Battalion) in 1910, and was promoted to the rank of captain last year. A keen sportsman, he had played cricket for Derby County and Rugby football for Blackheath. He was the son of the late Dr. Bingham, of Alfreton, in Derbyshire.

The Indian Medical Service has lost another good officer by the death of Major James Woods, whose name appeared in the casualty list recently. An Edinburgh University man, he graduated in medicine and surgery in 1901. Major Woods was fortunate in seeing plenty of active service during his Indian career; thus, in Frontal operations during 1908 he was present at Mohmand, Matta, and Kargha, being mentioned in dispatches and awarded the medal and clasp for his services in the field. After various other experiences, Major Woods found himself in civil employ in the Punjab at the outbreak of war, and after mobilisation became attached to the 39th Garhwal Rifles, with whom he continued until his work was ended, at the age of thirty-eight, during the severe fighting of the past few weeks.

Amongst members of the nursing profession who have heroically laid down their lives for their country must be mentioned Miss Phillis Pearse, of the Queen Alexandra's Imperial Military Nursing Service. Acting-sister Pearse, who was a daughter of L. F. Pearse, Esq., and only twenty-eight years old, was on her way home from Rouen when she died at Havre.

Lieutenant Arthur Cecil Hincks, who has been awarded the Military Cross, attracted special attention by his heroism at Neuve Chapelle, where, after being knocked senseless by a shell and his ambulance disorganised, he quickly pulled himself together, collected his wounded and by sheer strength of will conducted his work to a satisfactory end. A student of Birmingham University, Mr. Hincks qualified M.B. Ch.B. there in 1906, and after holding resident appointments at the Birmingham General Hospital, settled in practice at Wells, Somerset. A keen ambulance officer, he has been serving with the 26th Field Ambulance, Royal Army Medical Corps, Territorial Force (Wessex).

The Canadian medical profession has lost a capable officer by the death from wounds of Captain G. C. Glidden, which occurred recently. He had been serving with the West Canada Regiment (10th Battalion).

There can be no doubt that in the great *Lusitania* disaster both medical officers of the unfortunate ship perished miserably. They were Drs. James Farrell McDermott and Joseph Garry. As is often the case, a number of medical men were travelling on the liner in addition to the medical staff, and of these at least one, Dr. Owen Kenan, lost his life.

Both the medical officers on the *Lusitania* graduated in the Irish schools. Dr. James Farrell McDermott studied at Queen's College, Cork, and, taking the M.B., B.Ch. Irel. in 1906, subsequently joined the service of the Cunard Steamship Company; whilst Dr. Joseph Garry qualified M.B., B.Ch. Irel. only last year.

Dr. Thomas Grainger Stewart, who is taking over the direction of the massage and electro-therapy department at the King George Hospital, where he will control work of a most responsible kind, is physician to the Hospital for Paralysis and Epilepsy in Queen Square, as well as a member of the staff of the West London Hospital. As a neurologist Dr. Grainger Stewart has made important observations on the structure and pathology of the nervous system, particularly in regard to tumours of the brain. Studying medicine at Edinburgh University, he graduated M.B., Ch.B. there is 1900, and some time afterwards proceeded to the M.D. examination, at which he was awarded the gold medal. He was elected to the dignity of the Fellowship of the Royal College of Physicians of London two years ago.

The consulting staff of the Queen Mary's Convalescent Auxiliary Hospitals at Roehampton will consist of Dr. T. J. Horder and Dr. Theodore Thompson as consulting physicians, with Mr. Openshaw, Mr. Robert Jones, and Mr. R. C. Elmslie as consulting surgeons.

Dr. Thomas Jeeves Horder, M.D., F.R.C.P., is physician to the Great Northern Central Hospital, and is generally expected to succeed to the next medical vacancy on the staff of St. Bartholomew's Hospital, where he has carried out much excellent work during the past few years. A keen pathologist and bacteriologist, as well as clinician, Dr. Horder has made valuable contributions in regard to therapeutic inoculation.

Dr. Theodore Thompson is a London Hospital man, who has done good work in connection with the investigation of chest problems. After a brilliant academic career both at Cambridge and the "London," in the course of which he won many prizes and scholarships, and came out sixteenth on the list of Wranglers for 1899, he held various important junior posts at his hospital, and to-day occupies the position of physician to that institution. Dr. Theodore Thompson, who has many letters after his name—M.A., M.D., F.R.C.P. Lond., F.R.C.S. Eng., B.Sc. Lond.—was at one time physician to the Poplar Hospital.

Mr. Reginald Cheyne Elmslie, M.S. Lond., F.R.C.S. Eng., is a leading authority on orthopædic surgery. After a distinguished career at "Bart.'s," in the course of which he won the Lawrence scholarship and was awarded the gold medal at the London M.S. examination, he was appointed orthopædic surgeon at St. Bartholomew's. His present posts also include that of assistant surgeon to the Metropolitan Hospital.

